# Experimental study of histological changes in vascular loops according to the duration of the postoperative period: Application in reconstructive microsurgery

**DOI:** 10.6061/clinics/2017(09)03

**Published:** 2017-09

**Authors:** Renata Gregorio Paulos, Bruno Alves Rudelli, Renee Zon Filippe, Gustavo Bispo dos Santos, Ana Abarca Herrera, Andre Araujo Ribeiro, Marcelo Rosa de Rezende, Teng Hsiang-Wei, Rames Mattar-Jr

**Affiliations:** Departamento de Ortopedia e Traumatologia, Instituto de Ortopedia e Traumatologia (IOT), Hospital das Clinicas HCFMUSP, Faculdade de Medicina, Universidade de Sao Paulo, Sao Paulo, SP, BR

**Keywords:** Vascular Loop, Intimal Hyperplasia, Vascular Remodeling

## Abstract

**OBJECTIVES:**

To analyze the histological changes observed in venous grafts subjected to arterial blood flow as a function of the duration of the postoperative period to optimize their use in free flap reconstructions.

**METHOD:**

Twenty-five rats (7 females and 18 males) underwent surgery. Surgeries were performed on one animal per week. Five weeks after the first surgery, the same five animals were subjected to an additional surgery to assess the presence or absence of blood flow through the vascular loop, and samples were collected for histological analysis. This cycle was performed five times.

**RESULTS:**

Of the rats euthanized four to five weeks after the first surgery, no blood flow was observed through the graft in 80% of the cases. In the group euthanized three weeks after the first surgery, no blood flow was observed in 20% of the cases. In the groups euthanized one to two weeks after the first surgery, blood flow through the vascular loop was observed in all animals. Moreover, intimal proliferation tended to increase with the duration of the postoperative period. Two weeks after surgery, intimal proliferation increased slightly, whereas strong intimal proliferation was observed in all rats evaluated five weeks after surgery.

**CONCLUSION:**

Intimal proliferation was the most significant change noted in venous grafts as a function of the duration of the postoperative period and was directly correlated with graft occlusion. In cases in which vascular loops are required during free flap reconstruction, both procedures should preferably be performed during the same surgery.

## INTRODUCTION

Arteriovenous anastomosis is a procedure performed by various medical specialties and represents an optional treatment for different diseases. This procedure is used for the creation of fistulas in patients with chronic renal failure who require dialysis 1, in coronary artery bypass graft procedures, in free flap reconstruction 2,3, and for the repair of traumatic arterial lesions in general, including reimplantation and emergency revascularization.

One of the most important complications of this procedure is graft thrombosis, which may lead to treatment failure 4. Sterpetti et al. 4 reported that the rates of thrombosis of arteriovenous anastomoses one year after the procedure varied between 20% and 50%. One of the factors that may lead to such complications is intimal hyperplasia. This cellular proliferation phenomenon, which is also known as graft arterialization, is due to the increased pressure gradient in the graft after anastomosis.

The aim of this study was to analyze the histological changes observed in venous grafts subjected to arterial blood flow as a function of the duration of the postoperative period and therefore optimize their use in free flap reconstructions.

## MATERIALS AND METHODS

A total of 25 Wistar rats underwent surgery (7 females and 18 males), with a mean weight of 344 (248–440) grams immediately before the procedure.

Anastomoses were performed by the same surgeon in a microsurgery laboratory under the supervision of technicians.

One animal per week underwent surgery. Five weeks after the first surgery, the same five animals were subjected to an additional surgery to assess the presence or absence of blood flow through the vascular loop and collect samples for histological analysis. The rats were euthanized after this second surgical procedure. This cycle was performed five times to obtain five distinct animal groups, each containing five animals. The first, second, third, fourth, and fifth groups were sacrificed one, two, three, four, and five weeks after the anastomosis, respectively.

The guidelines for euthanasia outlined by the Brazilian College of Animal Experimentation (Colégio Brasileiro de Experimentação Animal–COBEA) (1999) were followed. At the end of the surgical procedure and while the animals were still anesthetized, thiopental was administered intraperitoneally to induce deep anesthesia followed by euthanasia through intracardiac administration of potassium chloride.

The samples collected for histological analysis included the left carotid artery containing the venous graft and the deep branch of the left jugular vein (the vein contralateral to that used as the graft served as a control for the evaluation of histological changes in the graft).

The samples obtained from each animal were identified, transferred to a test tube containing formalin, and used to prepare the histological slides. The samples were fixed in buffered 10% formalin and subjected to histological analysis. The vascular segment was fixed without sectioning. Five-micrometer histological sections were prepared and stained with hematoxylin and eosin using standard protocols. The histological analysis involved the semi-quantitative evaluation of the following variables: inflammatory infiltrates, vascular proliferation, fibrosis, intimal proliferation, and foreign body giant cell reactions. The semi-quantitative evaluation involved scoring the intensity of these variables using a scale ranging from 0 to 3, such that 0 indicated no intensity, 1 indicated mild intensity (1%–30%), 2 indicated moderate intensity (30%–60%), and 3 indicated severe intensity (greater than 60%). The presence of thrombi and pseudoaneurysms was also analyzed in the anastomosis area.

The inflammatory infiltrates had a mixed pattern and primarily contained neutrophils, lymphocytes, and plasma cells. Quantification, which was classified as mild, moderate, or severe, was determined by measuring the percentage of inflammatory infiltrates present in the anastomosis area. The degree of intimal proliferation was assessed according to the degree of intimal hyperplasia and the degree of protrusion into the vascular lumen. Foreign body giant cell reactions, i.e., reactions around foreign bodies that involve histiocytes and giant cells and that from granulomas, was evaluated using the same criteria used for the inflammatory infiltrates. The quantification of fibrosis (hyalinized fibrosis and reactive fibroplasia) and vascular proliferation (proliferation of the capillaries in the wall of the animal) was assessed by calculating the percentage of fibrosis and proliferation in the wall of the anastomosis area.

### Surgical procedure

Anesthesia was performed through intramuscular administration of 10 mg/kg xylazine and 50–75 mg/kg ketamine. After the animals were anesthetized, they were placed in the supine position, and then the skin was shaved and prepped with a local antiseptic and anesthetic.

A transverse incision at the base of the neck (shoulder to shoulder) was used as the access route. The trifurcation of the right external jugular vein was identified, and then its deep branch (mandibular) was dissected until the bifurcation was reached and connected at both ends. The distal end of the mandibular branch was sectioned, and the vessel lumen was irrigated with saline to prevent thrombus formation in the lumen.

On the contralateral side, the left carotid artery was identified and dissected. A surgical drape was placed under the left carotid artery, and a double clamp was placed on this vessel.

The proximal end of the venous graft (the mandibular branch of the right external jugular vein) was sectioned and fully freed. The direction of the graft was reversed, and the graft was placed on the surgical drape next to the left carotid artery.

Two arteriotomies were conducted, and the vessel was flushed with saline. The venous graft was sutured to the arteriotomies using two end-to-side anastomoses with 10.0 nylon sutures, and six sutures were used for each anastomosis.

**Figure 1:**
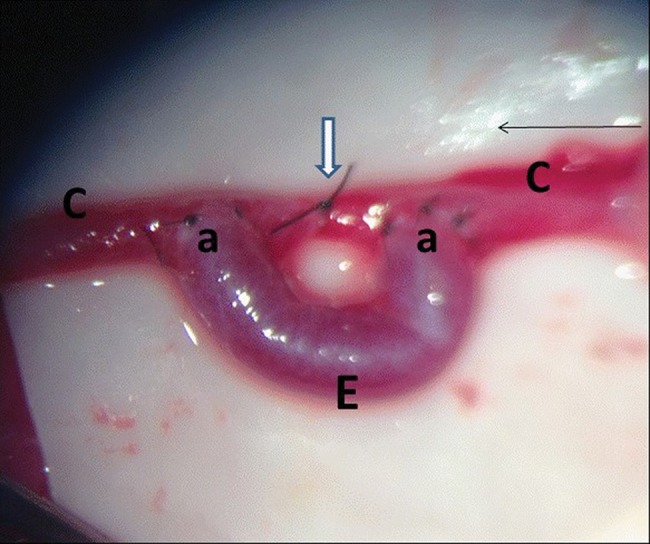
Final aspect of the surgical procedure. **C** – left carotid; **E** – venous graft; **a** – end-to-side anastomoses; small arrow – blood flow direction; large arrow – suture connecting the carotid artery to the end-to-side anastomoses.

Subsequently, an additional suture was used to connect the carotid artery to the two end-to-side anastomoses (Figure 1). The clamp was removed, and anastomosis patency was evaluated.

The wound was closed using simple non-absorbable wire sutures (nylon 4-0).

### Ehics

This study was approved by the Research Ethics Committee (number 043/14), and all animals were maintained in an animal facility with water and food ad libitum.

## RESULTS

In the first procedure, after the release of the clamp, adequate blood flow was observed in all cases.

In the group of rats sacrificed five weeks after the first surgery, blood flow was not observed through the graft in four animals (80%). A similar result was observed in the rats sacrificed four weeks after the initial surgery. In animals sacrificed three weeks after the initial surgery, blood flow was absent in only one animal (20%). In addition, blood flow was observed in the vascular loop in all animals in which the second surgery was conducted one to two weeks after the first surgery (Table 1).

In rat No. 25, blood flow was observed in the venous graft during the second surgery. However, the histological analysis indicated the presence of a thrombus in the vascular loop. In the remaining rats for which a thrombus was visualized during the histological examination, blood flow through the graft was not observed during the second procedure (Table 2).

**Figure 2:**
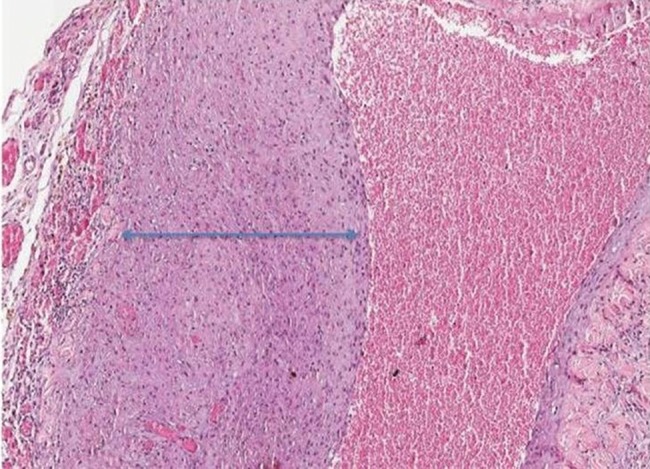
Slide showing strong intimal proliferation, which led to luminal stenosis (rat No. 24).

In the group sacrificed five weeks after the first surgery, the increase in vascular proliferation and intimal proliferation was moderate to severe in all animals evaluated (Figure 2). This group also presented the most significant changes in inflammatory infiltrates and fibrosis.

No pseudoaneurysms occurred in any of the cases evaluated, and granulomatous reactions to foreign bodies did not correlate with the duration of the postoperative period, i.e., they occurred at random.

The analysis by sex indicated that of the seven females and 18 males with follow-up, four females (57.15%) and five males (27.78%) exhibited no blood flow in the venous graft during the second procedure.

## DISCUSSION

Intimal hyperplasia is observed in venous segments after arteriovenous anastomoses. It is one of the leading complications associated with stenosis during coronary surgery 5 and leads to decreased blood flow and possible thrombosis 6. Intimal hyperplasia is considered the main cause of failure of arteriovenous fistulas.

Studies have been developed to elucidate the mechanism of venous graft wall thickening after the passage of arterial blood through the graft (a phenomenon known as arterialization). Numerous authors have designated the progressive changes that lead to obstruction as “venous graft disease.” These studies have primarily been conducted by cardiac surgery teams to prevent bypass graft loss 7-10.

The results observed in our study corroborate the findings of previous studies. We observed an increase in intimal proliferation in seven of the eight histological analyses of rats with failure of the vascular loop (one rat without blood flow could not be evaluated histologically due to a problem during the preparation of the slide).

We also noted that intimal proliferation tended to increase as the postoperative period increased. In two weeks, only a slight increase in the intima was observed, whereas intimal proliferation increased in all rats evaluated after five weeks. Similar results were observed by Wong et al. 1, who conducted an experimental study where arteriovenous fistulas were created in rats and then collected for analysis at 7, 14, and 28 days postoperatively. In their study, significant changes were observed in the intima after seven days. Furthermore, significant hyperplasia was observed two weeks after surgery.

Other changes observed included inflammatory infiltrates, granulomatous reactions to foreign bodies, fibrosis, and vascular proliferation. Among these changes, vascular proliferation also increased during the postoperative period.

In the rats with 4 and 5 weeks of postoperative recovery, 4 of the 5 (80%) grafts were occluded in each group. However, inflammatory infiltrates and fibrosis were more evident in the 5-week group. This result suggests that graft occlusion may increase inflammation and fibrosis, but the opposite effect (i.e., that inflammation and fibrosis would contribute to graft failure) does not occur, as would be expected.

In our study, of the seven female and 18 male rats evaluated, four females (57.15%) and five males (27.78%) experienced complications in the vascular loop. In this respect, previous studies have demonstrated that women are more likely to develop an occlusion of saphenous vein grafts 11,12.

Several studies have evaluated the use of arteriovenous loops in free flap reconstructions 2,3,13-20. These loops can be used at a stage in which the loop and free flap reconstruction are conducted simultaneously or in two stages, where the arteriovenous loop is conducted first, and free flap reconstruction is performed during a second surgery. At present, a tendency exists for the use of a single surgery 3 because an increased rate of complications, including venous graft occlusions, has been observed when both procedures are performed separately 17,21.

Our study demonstrated complete patency of the venous grafts in the first two weeks after surgery, and the histological analysis indicated that significant intimal proliferation started to become apparent three weeks after surgery. These results corroborate the preference for the simultaneous performance of vascular loop and free flap reconstruction. We observed that the failure rate of venous grafts related to intimal proliferation increased with the duration of the postoperative period. Therefore, performing definitive surgery without changes in the intima is safer.

Overall, autonomization of flaps was observed two to three weeks after surgery, which is the period necessary for the release of pedicle flaps, such as cross-leg flaps 22. Therefore, in cases of venous graft occlusion, flap loss would not occur after this period in single-stage procedures (loop and free flap reconstruction).

It was not our aim to determine the period of venous graft occlusion in medical practice because the time intervals observed in previous studies did not correspond to the intervals found in humans. However, our study suggests that the simultaneous execution of both procedures is safer because the vessel that receives the flap does not exhibit histological alterations that cause occlusions over time.

We noted that intimal proliferation was the most significant change in venous grafts during the postoperative period and was directly correlated with graft occlusion. Therefore, our results suggest that in cases in which vascular loops are required for free flap reconstruction, both procedures should be performed during the same surgery.

## AUTHOR CONTRIBUTIONS

Paulos RG designed the study and performed the surgeries. Rudelli BA analyzed the data and prepared the manuscript. Filippe RZ is the pathologist responsible for all the histopathological examinations. Santos GB ensured that the study followed the guidelines of the Brazilian College of Animal Experimentation (1999) for approval by the Research Ethics Committee. Herrera AA edited the manuscript. Ribeiro AA collected the data and created the tables. Rezende MR revised the manuscript. Hsiang-Wei T supervised the surgical technique and prepared the manuscript. Mattar-Jr R prepared and revised the manuscript.

## Figures and Tables

**Table 1:** Animals and changes observed.

Rat No.	Sex	Initial weight	Period until euthanasia	Blood flow through the vascular loop during the second surgery	Inflammatory infiltrates	Granulomatous reaction to foreign bodies	Fibrosis	Vascular proliferation	Intimal proliferation	Thrombus	Pseudoaneurysm
1	Male	350	5	Present	2	2	1	2	2	Absent	Absent
**2**	**Female**	**304**	**Died**								
3	Female	248	3	Present	1	2	1	1	1	Absent	Absent
4	Male	397	2	Present	1	2	1	2	1	Absent	Absent
5	Female	238	1	Present	1	1	1	1	1	Absent	Absent
6	Male	372	5	Absent	2	2	2	2	2	Present	Absent
7	Male	400	4	Absent	Slide not evaluated						
8	Male	415	4	Present	1	1	1	2	2	Absent	Absent
9	Male	435	3	Present	1	1	1	2	1	Absent	Absent
10	Male	381	2	Present	0	1	1	1	1	Absent	Absent
11	Male	440	1	Present	1	1	0	1	0	Absent	Absent
12	Female	295	5	Absent	1	1	2	2	2	Present	Absent
13	Female	280	4	Absent	2	2	1	2	2	Present	Absent
14	Female	293	3	Present	1	2	1	1	2	Absent	Absent
**15**	**Female**	**272**	**Died**								
16	Male	367	1	Present	1	1	0	1	0	Absent	Absent
17	Male	355	5	Absent	2	1	1	2	2	Present	Absent
18	Male	380	4	Absent	1	2	1	1	2	Present	Absent
19	Male	340	3	Absent	2	1	0	1	2	Present	Absent
20	Male	330	2	Present	1	1	0	1	1	Absent	Absent
21	Male	405	2	Present	1	1	1	1	1	Absent	Absent
22	Male	370	1	Present	0	1	1	1	1	Absent	Absent
23	Female	300	5	Absent	1	1	2	2	2	Present	Absent
24	Female	295	4	Absent	1	1	2	2	3	Present	Absent
25	Male	355	3	Present	1	1	1	1	1	Present	Absent
26	Male	347	2	Present	Slide not evaluated						
27	Male	330	1	Present	1	1	0	1	1	Absent	Absent

**Table 2:** Histological evaluation of rats with occlusion of the vascular loop.

Rat No.	Sex	Initial weight	Period until euthanasia	Blood flow through the vascular loop during the second surgery	Inflammatory infiltrates	Fibrosis	Vascular proliferation	Intimal proliferation
6	Male	372	5	Absent	2	2	2	2
12	Female	295	5	Absent	1	2	2	2
13	Female	280	4	Absent	2	1	2	2
17	Male	355	5	Absent	2	1	2	2
18	Male	380	4	Absent	1	1	1	2
19	Male	340	3	Absent	2	0	1	2
23	Female	300	5	Absent	1	2	2	2
24	Female	295	4	Absent	1	2	2	3
